# Functional and perceptive differences between conventional and advanced ankle foot orthoses in community ambulators post-limb trauma: the injuries managed with advanced bracing of the lower extremity (IM ABLE) study

**DOI:** 10.3389/fresc.2024.1277509

**Published:** 2024-07-01

**Authors:** M. Jason Highsmith, Rebecca M. Miro, Michael Kartel, Anita Ramrattan, Angela Courtade, Jeffrey T. Heckman, Samuel L. Phillips, Shane R. Wurdeman, Thomas V. DiBello, Dwiesha L. England, Phillip M. Stevens, James H. Campbell, Michael J. Hyre, Jason T. Maikos, Owen T. Hill, Stephanie L. Carey

**Affiliations:** ^1^ School of Physical Therapy & Rehabilitation Sciences, Morsani College of Medicine, University of South Florida, Tampa, FL, United States; ^2^ Orthotic, Prosthetic & Pedorthic Clinical Services (OPPCS) Program Office, Rehabilitation & Prosthetic Services, (12RPS4) US Department of Veterans Affairs, Washington, DC, United States; ^3^ Orthotic, Prosthetic & Pedorthic Clinical Services (OPPCS), Physical Medicine & Rehabilitation, James A. Haley Veterans' Hospital, US Department of Veterans Affairs, Tampa, FL, United States; ^4^ Research and Development Services, James A. Haley Veterans' Hospital, US Department of Veterans Affairs, Tampa, FL, United States; ^5^ Southeastern Regional Amputation System of Care, (ASoC) Physical Medicine & Rehabilitation, James A. Haley Veterans' Hospital, US Department of Veterans Affairs, Tampa, FL, United States; ^6^ Department of Neurology, Morsani College of Medicine, University of South Florida, Tampa, FL, United States; ^7^ Hanger Institute for Clinical Research and Education, Austin, TX, United States; ^8^ Narrows Institute for Biomedical Research and Education, New York, NY, United States; ^9^ Prosthetics and Sensory Aids Services, (PSAS) New York Harbor Healthcare System, US Department of Veterans Affairs, New York, NY, United States; ^10^ School of Health Professions, College of Medicine, Health Science Center, University of Texas, San Antonio, TX, United States; ^11^ Department of Mechanical Engineering, College of Engineering, University of South Florida, Tampa, FL, United States

**Keywords:** carbon fiber, energy storing, extremity trauma, rehabilitation, orthotics, AFO, IDEO

## Abstract

**Introduction:**

Many military service members and civilians suffer from lower extremity trauma. Despite recent advancements in lower limb bracing technology, it remains unclear whether these newer advanced braces offer improved comfort and functionality compared to conventional options. The IDEO (Intrepid Dynamic Exoskeletal Orthosis), a type of “advanced” orthosis was developed to assist in maintaining high functional performance in patients who have experienced high-energy lower extremity trauma and underwent limb salvage surgeries.

**Methods:**

A cross-sector multi-site initiative was completed to study the efficacy of advanced ankle foot orthoses (AFO) for lower limb trauma and injury compared to a conventional AFO. Following fitting, training, and accommodation, the subjects were assessed in each AFO system for mobility, self-reported function, safety and pain, and preference.

**Results:**

They preferred the advanced over the conventional AFO and the mobility and exertion perception improved with the advanced AFO with no difference in pain or overall health status scores.

**Discussion:**

Thus, an advanced AFO is an option for trauma affecting the lower limb. Long-term studies are required to better understand the accommodation and learning process of using an advanced AFO.

## 1 Introduction

It is estimated that 25,000–35,000 extremity trauma cases occur annually in the private sector (1). Additionally, the wars in Iraq and Afghanistan yielded approximately 20,000 extremity trauma cases. Limb salvage surgeries following lower limb trauma often result in orthotic utilization. Orthotic device options in such cases include conventional ankle foot orthoses (AFOs) and more recently, advanced AFOs such as the IDEO (Intrepid Dynamic Exoskeletal Orthosis) (1). The IDEO was developed to assist in maintaining high functional performance in patients who have experienced high-energy lower extremity trauma and underwent limb salvage surgeries. A recent systematic review of the effect of the IDEO concluded with four empirical evidence statements. Briefly, the four evidence statements were as follows:

1. In service personnel under 40 years of age, injured with high-energy lower extremity trauma, potentially confounded by posttraumatic ankle osteoarthritis, fitting, and the use of IDEO with return-to-run (RTR) physical therapy following limb salvage surgery may allow a return to active duty for a limited population of high-functioning patients.
2. In service personnel under 40 years of age, injured with high-energy lower extremity trauma, potentially confounded by posttraumatic ankle osteoarthritis, fitting, and the use of IDEO with RTR physical therapy following limb salvage surgery may allow a return to exercise, recreation and physical activity, and decreased pain for a limited population of high-functioning patients.
3. In service personnel under 40 years of age, injured with high-energy lower extremity trauma, fitting, and the use of IDEO with RTR physical therapy following limb salvage surgery results in improved agility, power, and speed, compared with no-brace or conventional bracing alternatives.
4. IDEO strut stiffness should be considered with respect to patient preference.

It is unclear if these results are more broadly applicable beyond younger service members with war and other military-related trauma. For instance, it is unknown if the benefits younger service members experience with limb salvage and IDEO use applies to Veterans who may also have experienced non-military trauma, later in their life following military service. It is also unclear if the results of the IDEO review apply to other non-IDEO energy storing and return advanced orthoses.

Beyond the dearth of AFO outcome literature in limb trauma and advanced AFO space, prosthetic and orthotic literature on device training and accommodation is also limited. Clinical trials on prosthetic knees identified the importance of controlling for, assessing, and measuring these phenomena. Further, the subject has been editorially described as it relates to prostheses, yet comparable literature seems notably absent as it relates to orthotic science and practice. Therefore, standardized accommodation and training protocols and their effect on outcomes are largely unavailable and unknown (1–3).

Given the lack of outcomes comparing AFO types, the lack of clinical guidance, and the lack of training and accommodation evidence, the IM ABLE (Injuries Managed with Advanced Bracing for Lower Extremities) study, a multi-site, multisector initiative that included collaboration between VA, the military, and private sectors and included experts in physical therapy, orthotics, rehabilitation medicine, and engineering was developed. The primary objective of the IM ABLE clinical trial was to determine if training with and use of advanced (ADV) AFOs would lead to improved mobility, self-reported function, safety and pain, and preference for them compared with conventional (CONV) AFOs for those ambulating at or above the independent community level of ambulation following limb trauma.

## 2 Materials and methods

Three clinical sites were involved in the study, namely, (1) James A. Haley Veterans' Hospital (VAMC—Tampa, FL, USA); (2) VA New York Harbor Healthcare System (New York, NY, USA); and (3) Hanger Clinic (Houston, TX, USA) with The University of South Florida (USF) School of Physical Therapy and Rehabilitation Sciences serving as the data coordinating center. The study was approved by the following Institutional Review Boards (IRBs): VA Central IRB, Western IRB, and USF IRB. Additional approval was provided by the Human Research Protections Office (HRPO, US Army), and the study was listed in the US Federal Clinical Trials Registry (ClinicalTrials.gov registry: NCT03107728). A randomized, multi-site, two-period cross-over study design was utilized. Both the objective and subjective functions of conventional ankle foot orthosis (CONV AFO) were compared with advanced ankle foot orthosis (ADV AFO). AFO device allocation was randomly assigned off-site via computer number generation and concealed from the study PT until device training and from investigators until data analysis to improve methodologic quality and minimize bias risk. The study statistician was also blinded to the data until the final review and analysis were complete. Greater protocol details are published elsewhere (2).

### 2.1 Participants

Participants who have experienced lower extremity injury requiring the use of either an ADV or CONV AFO were recruited. “Established patients,” meaning those already using an AFO, were recruited from the populations of the participating clinics. Patients with acute injury and who underwent rehabilitation and orthotic selection for the first time were not recruited due to strong confounding parameters from initial recovery, other medical interventions and therapies, and initial selection of device(s). The specific eligibility criteria were as follows:

Inclusion criteria:

1. Lower extremity injury of any etiology requiring the use of an AFO
2. Any gender or ethnicity
3. 18–65 years of age
4.  100–275 lbs
5. >1 year of orthotic experience

Exclusion criteria:

1. <18 or >65 years of age
2. Body weight <100 or >275 lbs
3. <1 year of orthotic experience

### 2.2 Accommodation and training

**Figure 1:**
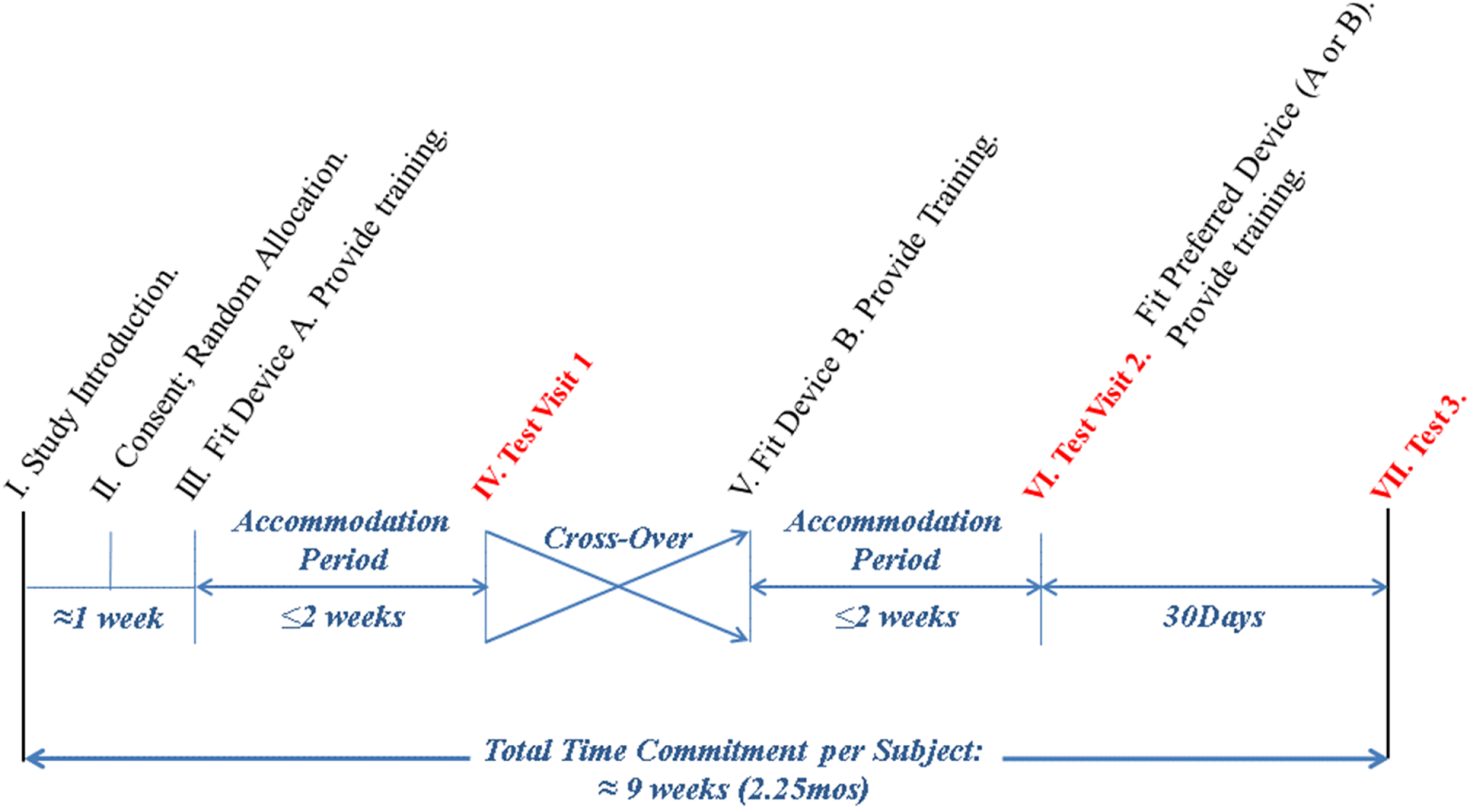
Study flow diagram.

Once enrolled, the subjects were randomized to either continue using their established AFO (ADV or CONV) or to begin the first arm of the study with the alternative AFO that they did not have. Following accommodation with the initial device, the process was repeated once subjects crossed over into the second arm of the study to use the alternate device. Briefly, the subjects were cast and fit with the alternative device and then received AFO training. In both arms of the study, they were tested for accommodation with the respective AFO device prior to being tested with it. The accommodation period was up to 2 weeks. However, the participants were able to verbally confirm that they were ready to test at any point during the 2-week period to demonstrate accommodation. After the accommodation period, the participants were assessed to confirm accommodation, and data collection was completed. More details regarding inclusion, exclusion, and discontinuation criteria have been previously published (2). Figure 1 shows the total time commitment and the data collection time for the subjects.

### 2.3 Outcome measures

Several measures were collected to compare the CONV and ADV AFOs. Following fitting, training, and accommodation, the subjects were assessed in each AFO system for mobility, self-reported function, safety and pain, and preference. Functional mobility performance was measured by the “timed up and go” (TUG) test and the 2 min walk test (2MWT). The self-reported function was measured by the Borg rating of perceived exertion immediately following the 2MWT and the activity-specific balance confidence (ABC) scale. Included with self-reported measures was a pain assessment and the self-reported number of falls. These measures were completed twice, after the accommodation and training of the CONV and ADV AFOs.

#### 2.3.1 Mobility

##### 2.3.1.1 Timed up and go (TUG)

In the “timed up and go” test, the subjects are timed while they rise from a chair, walk 3 m, turn around, return to the chair, and sit down again. The patients are usually permitted to use a walking aid, but not physical assistance. The same standard chair should be used for all subjects (and sites; i.e., seat height, 47 cm; arm height, 67 cm). The patients are permitted a practice test before being timed, and the test is usually repeated three times. The TUG is a reliable and valid test for quantifying functional walking ability in elderly people with varied medical history, including stroke (3–5). Meta-analysis of data from 4,395 subjects from 21 studies showed homogeneity of times. Their overall mean time for the TUG (9.4 s) had a narrow confidence interval (8.9–9.9 s) (5). Normative TUG times are also available by age groups (60–69 years, 70–79 years, 80–99 years) and are shown to increase with increasing age: 8.1 s, 9.2 s, and 11.3 s respectively (6). Minimal detectable change (95% confidence interval) in persons with chronic stroke measured with the TUG has been reported to be between 4 s and 9 s or a change of 21%–30% depending on the degree of tone about their involved ankle (7). Another study showed time reductions of 1.4 s as indicative of “major improvement” in those seeking care for hip arthritis (8).

##### 2.3.1.2 2MWT

The 2MWT is a modified version of the 6MWT and is a measure of self-paced walking ability. It has been routinely used at the James A. Haley Veterans' Hospital since 2008 and is being considered more widely by the VA as a standard measure as part of the Amputation System of Care (9). The 2MWT is valid and reliable in multiple diagnostic groups including those with lower limb amputation, neurologic impairment, and others (10). In a sample of 1,137 subjects, the mean distance walked on the test was 180.9 m with a range of 64.6–300.8 m. The minimum detectable change for the 2MWT was reported to be 42.5 m in this healthy sample (11). Other MDC values have been reported in participants with lower limb amputation at 34.4 m, 22.9 m in those with poliomyelitis, and 13.4 m in those with stroke. Meta-analyses and reviews report improvements in walking and mobility as a result of AFO use. Using standard methods, the 2MWT was administered by asking the subjects to walk the greatest distance they could in 2 min while walking a 15.2 m “out and back” course until asked to stop (12). The distance walked was recorded. The subjects were allowed to stop and rest in standing or sitting during the test. No encouragement was given, and there was no talking during the test. The rater started the timer when the command “go” was given, and the timer was stopped after the subject walked 2 min. The subjects were allowed to use an assistive device, but not physical assistance. One trial was performed, with the distance recorded upon completion.

#### 2.3.2 Perceptive function: self-reported measures

##### 2.3.2.1 Borg rating of perceived exertion (RPE)

The participants were asked to rate perceived exertion using the Borg rating (13) after completing the 2MWT. The Borg RPE scale is a tool to measure how hard it feels to the subject to engage in a physical activity. The Borg RPE is a scale from 6 (no exertion at all) to 20 (maximal exertion). Upon completion of the test, the rater will ask the subject to rate their perceived exertion (RPE) level using the Borg scale (6, no exertion; 20, max exertion) before and after the test.

##### 2.3.2.2 ABC scale

The ABC scale allows for the self-rating of the degree of confidence in balance during activities of daily living. It has been used to determine balance confidence in the elderly (14) and with individuals with a lower limb amputation (15). The participants responded to 16 questions related to tasks such as walking around the house or climbing stairs with a 0%–100% continuous scale. Additionally, the ABC scale is a 16-item self-report measure of the perceived balance confidence an individual has while completing various ambulatory activities. The participants estimate on a scale of 0%–100% how confident they are at performing activities such as picking a slipper up from the floor or walking on a slippery surface without losing balance. The item scores are summed and divided by 16 to provide an overall mean balance confidence score. The assessment is valid and reliable in those with lower limb amputation, stroke, and other demographic groups (15, 16).

##### 2.3.2.3 Visual analog scale (VAS) numeric pain scale

The VAS is a validated and subjective measure of acute and chronic pain (17). The participants were asked to make a handwritten mark on a 10 cm line that represents a continuum between “no pain” and “worst pain.” A VAS score was completed by each participant after using the CONV and the ADV AFO describing the sound (uninvolved) limb, the limb with orthosis (involved), and the lower back.

##### 2.3.2.4 EQ-5D and health status questionnaire

The EQ-5D is a five-item, ordinally scaled patient-reported outcome assessment developed by the EuroQOL group to determine health-related quality of life (18). The EQ-5D is widely used and measures health status on five dimensions, namely, mobility (EQ1), self-care (EQ2), usual activities (EQ3), pain/discomfort (EQ4), and anxiety/depression (EQ5). Three associated response options (no problem, some problems, and extreme problems) are available for measuring health status on the five dimensions. This combination of items and responses produces 243 possible health states. There is also a visual analog scale that allows subjects to rate their overall health status from 0 to 100. This utility score was derived from preferences for 45 states and ranges from −0.594 to 1. The EQ-5D is the preferred instrument for use in submissions to the NICE appraisal process (19). EQ-5D has been used in multiple diagnostic groups including those with stable angina, mental health, and others (20, 21). The EQ-5D was used at each data collection to determine if the health state improved in those using AFOs.

#### 2.3.3 Preference

**Figure 2:**
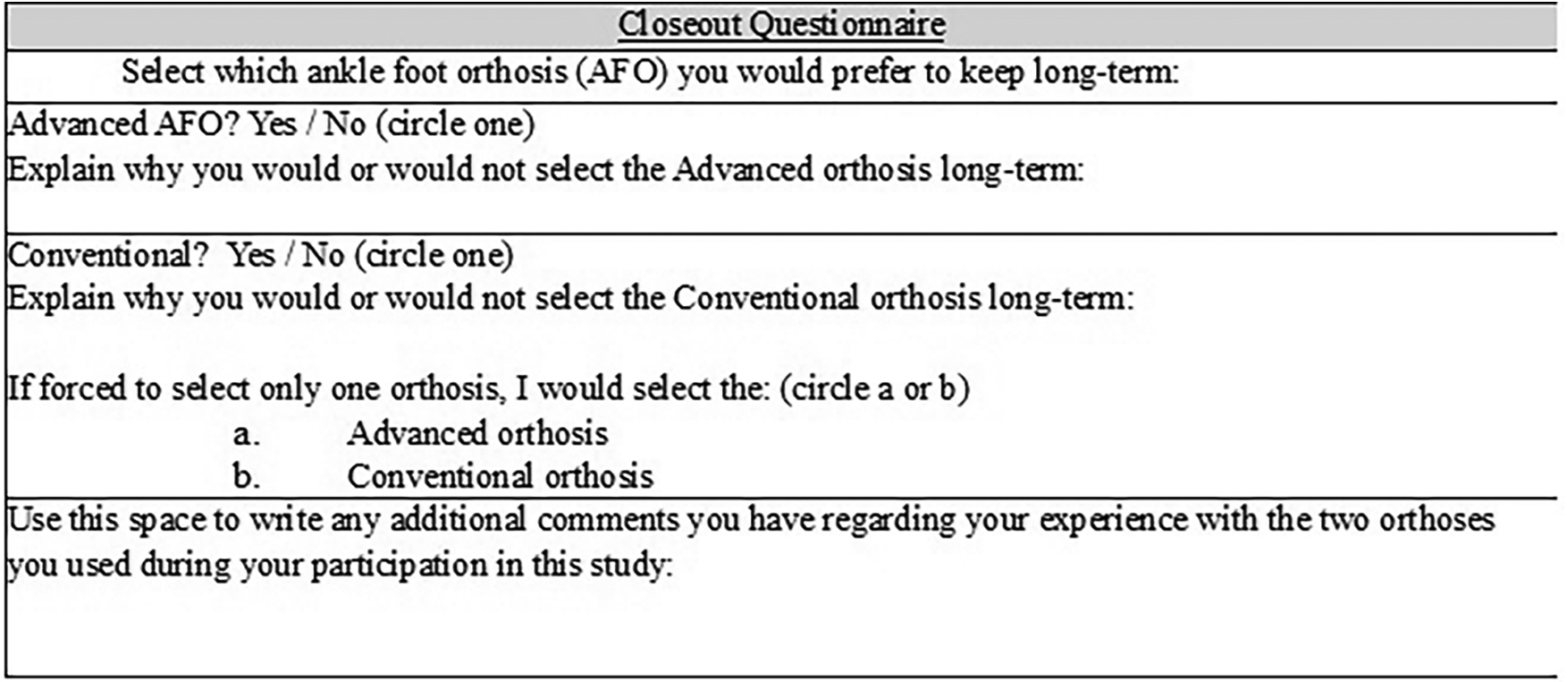
Closeout questionnaire.

Finally, an *ad hoc* closeout questionnaire addressed AFO preference. At the conclusion of the study, a closeout questionnaire was given to the subjects. Preference is a vital measure in users of assistive devices (22, 23). The participants were asked which orthosis was preferred to determine true user preference regardless of functional performance data. The subjects were asked the following three questions: (1) “Would you select the advanced (ADV) orthosis long-term?”, (2) Would you select the conventional (CONV) orthosis long-term?”, and “If you are forced to select only one orthosis, would you select the ADV or CONV orthosis.” Figure 2 depicts the closeout questionnaire, which was given to the subjects.

### 2.4 Data analysis

The primary hypothesis for the study was to determine if, compared to the standard of care CONV AFO design, the ADV alternative AFO design (independent variables) improved mobility, self-reported measures, and preference. To assess this, outcomes of interest included function as measured by TUG times, 2MWT distance, ABC scale scores, and preference and perceptive measures (dependent variables). In this study design, cross-over trials in which subjects use multiple devices (i.e., repeated measures), a comparison of means (presuming normal distribution) using a dependent sample *t*-test (or non-parametric equivalent such as the Wilcoxon signed-rank test) is standard practice. This enables a comparison of means between device types (ADV compared with CONV). The more traditional *t*-test method of analysis assumes complete data sets.

Data were entered into a database and verified for normality (for continuous variables—e.g., 2MWT distance, TUG times), completeness, and outliers prior to analysis. In cases where data were abnormally distributed, when appropriate, data were transformed accordingly. When transformations were unrealistic, data were adjusted by dichotomizing into subgroups (*post hoc*) of given outcomes (e.g., based on function, ability to complete a task or not, and type of component) All primary, *a priori* analyses compared ADV and CONV values relative to each other. Variables for *post hoc* analyses included age, BMI, time from injury, and etiology. Parametric statistics were used when possible, and non-parametric tests were used when needed (i.e., abnormal distribution, non-continuous, and ordinal data). For all analyses, a *p*-value of ≤0.05 was considered statistically significant. Fisher's exact test better for small samples was used to determine if there was a significant association between the two AFO categories (CONV, ADV) in a 2 × 2 contingency table. The investigators adopted the mean substitution method to deal with missing data from two subjects as the study's *a priori* intention-to-treat plan (24).

## 3 Results

### 3.1 Participant demographics and physical examination findings

A total of 76 subjects provided written informed consent and completed some portion of the study. From these, 58 AFO users (*n *= 58) completed all study tasks for both the CONV and ADV. Most subjects were male (75%) with a mean age (SD) of 52.3 years (15.2). The participants had a mean (SD) height of 173.8 cm (10.5) and a mean mass of 93.3 kg (22.0). Two subjects were bilaterally involved and used bilateral AFOs. Etiologies necessitating the use of an AFO were varied including a mix of neurologic, vascular, traumatic issues, and other diagnoses as shown in Table 1. Eleven subjects had a fused ankle. Most of the sample described themselves as retired (41%), whereas 19% self-reported to be unemployed. Many within these two categories were also governmentally classified as disabled. A considerable subset of the sample was employed; 21% were engaged in physically demanding work (i.e., chef, electrician, camp director, and landscaper), and 19% participated in office-related work (i.e., accountant, administrative assistant, lawyer, and IT analyst). All subjects were capable of community ambulation; 11% were limited community ambulators, 56% were unlimited community ambulators, and 33% of the sample participated in exercise and sport. The subjects reported an average of 1.5 ± 3.2 falls (range, 0–20) within the 12 months prior to their participation in the study.

**Table 1:** Etiological indication and need for AFO use. Neurologic injuries include cerebral palsy, Charcot–Marie–Tooth disease, Guillain–Barre syndrome, multiple sclerosis, poliomyelitis, post-polio syndrome, spina bifida, spinal cord injury, stroke, transverse myelitis, and traumatic brain injury.

Etiology/diagnosis	% of sample
Avulsion fracture	2%
Neurologic injury	64%
Drop foot	17%
Pain	6%
Surgical complications	2%
Trauma	11%

Initial physical examination by the study physical therapists revealed that 70% of subjects' upper extremities were rated as within normal limits (WNL) for strength as tested by manual muscle testing (MMT) and range of motion (ROM). Non-paralytic strength and ROM impairment were found in 17% of subjects' upper limbs, and 13% had some degree of paralytic impairment.

The ROM was impaired in a greater number (*p *= 0.001) of involved side joints compared to uninvolved side lower extremity joints (Table 2). The greatest ROM impairment was seen with involved side ankle dorsiflexion (96% of limbs) followed by hip abduction (72%), plantar flexion (70%), and hip flexion (64%). Dorsiflexion was the most impaired joint movement, in terms of ROM, on both sides.

**Table 2:** Percentage of limbs by side with a range of motion impairment.

Joint	Movement	Involved side	Uninvolved side
Hip	Flexion	64%	18%
	Abduction	72%	21%
Knee	Flexion	58%	17%
	Extension	10%	3%
Ankle	Dorsiflexion	96%	27%
	Plantar flexion	70%	20%
	Inversion	51%	15%
	Eversion	29%	8%

Regarding MMT, most participants' involved side limbs (70%–79%) were WNL (4–5/5) relative to the hip and knee. Considerably less involved side ankle movements (35%–45%) were WNL. Hip and knee movements were minimally impaired for strength relative to the ankle. Subjects' involved side ankles demonstrated a considerable percentage of strength impairment (6%–26%) (Table 3).

**Table 3:** Percentage of involved limb muscle impairment by joint movement. Manual muscle test (MMT) findings as measured manually by licensed physical therapists on a 0–5/5 (ordinal) scale. WNL, within normal limits. For this analysis, MMT scores of 4/5 or 5/5 were regarded as WNL and are shown here aggregated. Scores at 3/5 or below are shown at the respective score level. The values represent the percentage of involved side limbs within their respective score values.

Joint	Hip		Knee		Ankle			
Movement/MMT grade	Flexion	Abduction	Flexion	Extension	Dorsiflexion	Plantar flexion	Inversion	Eversion
WNL: 4–5	74%	75%	70%	79%	42%	35%	45%	42%
3	11%	11%	16%	16%	11%	15%	6%	8%
2	3%	5%	5%	4%	7%	13%	6%	14%
1	3%	1%	0%	0%	11%	9%	11%	7%
0	2%	1%	1%	1%	20%	18%	26%	26%

Spasticity of the ankle was graded during physical examination with the modified Ashworth scale (Table 4). Some degree of neurologic impairment both at the upper and lower motor neuron level was present in the sample. Therefore, spasticity was present. Most subjects did not have spasticity at the ankle. A slightly higher percentage of involved side ankles had spasticity compared to uninvolved side ankles; 23% compared with 9%, respectively. A timed, single-limb balance test was conducted with subjects not wearing their AFO(s). Subjects were able to balance longer (*p *< 0.0001) on the uninvolved (or stronger) side (10.3 ± 11.2 s) compared with the involved (or weaker) limb (3.7 ± 7.3 s).

**Table 4:** Percentage of subjects with spasticity as measured with modified Ashworth scale: 0, no increase in muscle tone. 1, slight Increase in muscle tone, manifested by a catch. 1+, slight increase in muscle tone, manifested by a catch followed by minimal resistance through the remainder of the range. 2, more marked increase in muscle tone, through most of the ROM, but affected part(s) easily moved. 3, considerable increase in muscle tone, passive movement difficult. 4, affected part(s) rigid in flexion/extension.

Spasticity assessed with modified Ashworth scale		
Grade	Involved ankle	Uninvolved ankle
0	61%	77%
1	8%	1%
1+	4%	0%
2	8%	8%
3	3%	0%
4	0%	0%

Nearly half of the sample (48%) reported pain at entry into the study. Of those, the two most identified body sources of pain were the involved (or weaker) leg and the back. (Table 5) The mean pain intensity was 5.7 ± 2.3 upon initial examination. Over a third (36%) of the sample were taking medications or using a device to manage their pain at the start of the study (Table 6). Eight subjects were using two to four drugs (or a device) simultaneously for pain management.

**Table 5:** Body source of pain on initial examination.

Pain source at initial physical examination		
Area	Primary	Secondary
Leg	22%	6%
Back	16%	17%
Shoulder	3%	0%
Hip	2%	0%
Groin	2%	2%
Ankle	2%	3%
Knee	0%	3%
Foot	0%	2%

**Table 6:** Percent of drug type among those taking medications or using devices to manage their pain.

Drug class or device	%
GABA analog	30%
Analgesic	26%
analgesic patches	22%
narcotic opioid	17%
Other	13%
Narcotic analgesic	9%
Serotonin and norepinephrine reuptake inhibitor	9%
GABA analog	4%
Muscle relaxant	4%
Non-steroidal anti-inflammatories	4%
Nerve stimulator	4%

In terms of training for the devices, subjects utilized 1.2 ± 0.4 training sessions with the CONV AFO compared with 1.3 ± 0.6 (range 1 to 4; *p *= 0.11) sessions with the ADV AFO prior to each respective data collection.

### 3.2 Orthotic design and utilization

Most of the sample (75%) entered the study originally using a CONV AFO and the remaining 25% with an ADV AFO. For subjects whose original prescription was a CONV AFO, the mean (SD) time with current CONV AFO was 2.8 (4.5) years. For those whose original prescription was an ADV AFO, the mean time (SD) with the ADV AFO was significantly shorter (*p *< 0.001) at 0.3 (0.7) years.

For original CONV AFO users, the mean (SD) number of AFOs that subjects recalled using prior to the study was 3.7 ± 3.5. For AFO users who participated in the study originally using an ADV AFO, the mean (SD) number of AFOs used (by recall) prior to the study was 4.1 ± 3.3. All subjects recalled using an average of 4.0 ± 3.5 (range, 0–20) AFOs prior to the study.

From the sample, 29 subjects used an assistive device for ambulation and transfers and the AFO had no effect on changing, which assistive device was used. Some type of cane (single, two, three, or four points) was used by 22 subjects, 6 used a walker, and 1 used a wheelchair. Two subjects utilized two assistive devices.

### 3.3 AFO construction

Sixty-three percent of CONV AFOs were primarily constructed with thermoplastic materials and were fully custom fabricated. Two were laminated finish, and 13 were fabricated with various other materials or combinations of thermoplastics and laminate materials. Fourteen were off-the-shelf designs. Thirteen CONV AFOs were articulated, and four incorporated a strut mechanism in their design. Seventeen of the CONV AFOs included plantar flexion in their design and alignment. Five of the CONV AFOs were posted with some form of a wedge. Twenty-four CONV AFO users were unable to wear their preferred footwear, and four CONV AFOs reportedly caused skin issues. Ten CONV AFOs caused pain that was rated 2–10 with a mean rating of 7/10 intensity. When using the ADV AFO, the same 10 subjects also reported pain ranging from 1 to 10 with a mean intensity of 7/10 (*p *> 0.05). Of note, 53 participants did not report pain associated with AFO use. All but four CONV AFOs included strapping secured with Velcro. Two were secured with laces, one with a BOA system and one with the combination of a BOA system and Velcro strapping. In this sample, all subjects except two were able to independently and efficiently don the CONV AFO and their respective footwear.

All ADV AFOs were fully custom. Ten were not plantarflexed, and 16 were posted with a mediolateral wedge, all with a loose wedge technique. Five ADV AFOs reportedly caused skin issues. Forty subjects were reportedly able to wear their preferred footwear with the ADV AFO. The ADV AFOs were not articulated with a joint in the usual manner that a CONV AFO may be as they all incorporated an energy-storing (carbon fiber construction) strut assembly, which through bending allowed a pseudo-articulation. Sixty subjects reported the ability to independently don the ADV AFO, whereas three subjects could not. Fifty-five ADV AFOs incorporated Velcro strap closure, six used a BOA closure system, and one had a combination of Velcro and a BOA system.

Finally, the actual size of the devices was compared in terms of trimline height and toe plate length. The relative trimline build height was compared by determining the percent difference between the groups' tibial tubercle to floor length and the maximal AFO trimline height to the floor (in cm then converted to a percentage). The CONV AFO trimline height was 88% ± 12%, which was significantly shorter than the ADV AFO which was 93% ± 4% (*p *= 0.004). That is, the ADV AFO is 93% of the distance from the floor to each subject’s tibial tubercle. A similar observation was found in the percentage of the toe plate relative to the foot length. The CONV AFO toe plate was on average, 93 ± 9% of the length of the patient's foot, whereas the ADV AFO was on average, 96% ± 4% of the length of the patient's foot (*p *= 0.008).

### 3.4 Mobility results

The results from the functional performance test: the TUG and the 2MWT are shown in Table 7. While using the ADV, there were no significant differences for the TUG or the 2MWT.

**Table 7:** Outcome measurements with *p*-values.

		CONV (AVG ± SD)	ADV (AVG ± SD)	% Difference	Two-sided *p*-value
Mobility					
	TUG (s)	14.9 ± 12.0	16.5 ± 16.4	+10.2%	0.281
	2MWT (m)	97.8 ± 37.7	101.7 ± 42.4	+ 4.0%	0.257
Perception					
	RPE Borg scale (6–20)	11.7 ± 3.1	10.8 ± 3.0	**-8.0%**	.015*
	ABC scale %	63.4 ± 21.7	65.3 ± 22.8	+3.0%	.422
Pain					
	VAS pain scale: involved	3.2 ± 2.8	3.2 ± 3.0	0%	.950
	VAS pain scale: uninvolved	1.6 ± 2.5	1.8 ± 2.5	+11.8%	.433
Health Status				** **	** **
	EQ1: mobility	1.7 ± 0.5	1.6 ± 0.5	**−6.1%**	.037*
	EQ2: self-care	1.4 ± 0.5	1.3 ± 0.5	−7.4%	.264
	EQ3: activities	1.6 ± 0.6	1.6 ± 0.6	0%	.558
	EQ4: pain/discomfort	1.7 ± 0.6	1.7 ± 0.5	0%	.371
	EQ5: anxiety/depression	1.5 ± 0.6	1.5 ± 0.6	0%	.386
	EQ-5D health rate	70.4 ± 19.8	71.4 ± 20.2	+1.4%	.685

Values bold font and * indicate a statistically significant difference.

### 3.5 Perceptive functional performance results

The results from the perceptive functional performance: the RPE during the 2MWT and the ABC scale are shown in Table 7. While using the ADV the participants perceived the 2MWT to be less challenging. There were no significant differences in the ABC score between CONV and ADV use.

### 3.6 Pain and health Status results

Table 7 shows the VAS numeric pain scale and the EQ1-5 results by question and the overall health status. There were no differences in pain or perceived overall health status when using the CONV or ADV device. Table 8 shows the EQ5 score describing the perception of mobility and a greater percentage of subjects experiencing no mobility problems with the ADV device.

**Table 8:** EQ5: mobility.

	CONV	ADV
Level 1: no problems	31%	45%
Level 2: some problems	68%	55%
Level 3: extreme problems	2%	0%

### 3.7 Preference

The subjects were asked if they would use the advanced AFO long term and if they would use the conventional AFO long term. Table 9 shows the 2 × 2 frequency table used for Fisher's exact Test. Fisher's exact two-tailed test had a *p*-value of <0.0001, suggesting that users preferred the advanced AFO over the conventional AFO when considering long-term use. When subjects were asked to choose one AFO for overall preference, 80% of the subjects preferred the ADV AFO overall.

**Table 9:** 2 × 2 frequency table for Fisher's exact test.

	Yes	No	Total
ADV	48	10	58
CONV	14	45	59
Total	62	55	117

## 4 Discussion

Recent reviews of the literature (25) have described types of AFOs and the clinical trials exploring the impacts of these AFOs on stroke recovery, peripheral artery disease, multiple sclerosis, cerebral palsy, and traumatic conditions. Fatone et al. (26) completed a review to identify instruments to assess care quality for individuals with custom AFOs that recommended using the TUG as a quality measure for AFO care for persons with neurologic or traumatic conditions.

Previously reported TUG times for stroke patients using an AFO have a mean ranging from 23.4 to 31.3 s (27). The participants in this study of younger aged patients with more heterogeneous diagnoses completed the TUG faster than those with stroke with 14.9 s completion times when using a CONV AFO and 16.5 with an ADV AFO.

Normative mean walking distances on the 2MWT derived from a meta-analysis for older adults residing in long-term care were 77.4 m, those with lower limb amputations walked 27.9 m, those with chronic stroke walked 58.5 m, those with late-onset sequelae of poliomyelitis walked 136.0 m, those with cardiac disease walked 138.0 m, and those with chronic obstructive pulmonary disease (COPD) walked 150.0 m (28). A population with a unilateral transfemoral amputation averaged 132 m (29) on the 2MWT. For comparison, this sample of AFO users walked 97.8 m (CONV) and 101.7 m (ADV). (Table 7) This sample walked farther than those from other studies of chronic stroke but not as far as those with late-onset sequelae of poliomyelitis, cardiac disease, or COPD. Lower extremity amputation studies analyzed gait with such outcomes as the TUG and 2MWT are more prevalent than for comparing AFO use. Having comparative performance information about multiple diagnoses is useful. However, having data on both lower limb prosthetic and orthotic use will help patients, caregivers, and clinicians in limb salvage and amputation decisions.

The IDEO, the advanced AFO often prescribed to service members who tend to be younger with more traumatic-related issues, has been shown to improve physical performance (the four-square step test, timed stair ascent, self-selected walking velocity, and the 20 m shuttle run) and patient-reported outcomes [short musculoskeletal function assessment (SMFA), the Veterans Rand 12-item Health Survey (VR-12), and the visual analog pain scale (VAS)] (29). The subject sample reported here did not experience the same magnitude of these objective benefits as did with a younger (<40 years old) military population (30).

This sample here was different in terms of older age, more neurologic impairment, and pain. Unlike a military population who perform rehabilitation as part of duty and service, this sample had the choice and autonomy to participate in training and therapy and opted for less training, which may have yielded less accommodation time and potentially confounded performance due to not being fully accommodated. The ADV device is physically larger, which has benefits in terms of a lever arm for dynamic elastic response/energy storing and return. This may provide advantages in terms of prolonged steady-state walking (2MWT, RPE) but can potentially confound smaller, confined space, transitional movement, and turning maneuvers such as an increased TUG time. Regardless of etiology that varied and included upper motor neuron, lower motor neuron, pain, and orthopedic trauma, the subjects were still able to train with, successfully use, and ultimately experience decreased exertion, fewer mobility issues, and a preference for advanced AFOs.

This study's main limitation was due to the recruitment and retention of research participants. This study was interrupted by the COVID pandemic when hospitals and clinics were closed for the most non-life-threatening treatment. When research recruitment and testing resumed, it was limited, and the subject pool may have been skewed due to self-selection bias. This may have caused the study to be underpowered for physical performance measures.

The study team is in the process of developing a follow-up study. It will include the use of activity monitors embedded into the AFOs, additional outcome measures such as the four-square step test, Patient-Reported Outcomes Measurement Information System (PROMIS), Quebec User Evaluation of Satisfaction with assistive Technology (QUEST 2.0), and Orthotics and Prosthetics Users' Survey (OPUS). After training and accommodation, the subjects will have both conventional and advanced AFOs to use over the course of a year. Long-term evaluation and economic analysis will be completed.

## 5 Conclusion

Overall, the subjects perceived that the ADV AFO increased mobility, required less effort, and was preferred over conventional alternatives. While in this population, the mobility measures such as TUG and 2MWT did not show significant differences, a longer-term study, with increased emphasis on training and accommodation, may be necessary to understand the length of time and volume of training necessary to see actual significant functional performance changes beyond subject perception. Given that there were no detriments to skin, pain, or health status, that functional performance was not different, and that subjects perceived less exertion, had fewer mobility issues, and preferred the ADV device, ADV AFOs in comparable patients should be considered as an option. Moreover, in patients who are challenged by increased exertion with steady-state walking and with mobility issues when using a CONV AFO, the ADV AFO may be a superior alternative to consider.

## Data Availability

The de-identified raw data supporting the conclusions of this article will be made available by the authors, without undue reservation.
